# Association Between Adherence to a Locally Adapted Enhanced Recovery After Surgery Pathway and Perioperative Outcomes After Open Abdominal Aortic Aneurysm Repair: A Retrospective Cohort Study

**DOI:** 10.3390/jcm15166486

**Published:** 2026-08-21

**Authors:** Zhiyi Yang, Qinghe Wang, Qingfeng Li, Xinyu Cheng, Yutong Liu, Jing Cai, Tong Qiao

**Affiliations:** Department of Vascular Surgery, Nanjing Drum Tower Hospital, Affiliated Hospital of Medical School, Nanjing University, Nanjing 210008, China

**Keywords:** ERAS protocol, abdominal aortic aneurysm, perioperative, open surgical repair

## Abstract

**Objective:** Open surgical repair (OSR) remains an important treatment for abdominal aortic aneurysm (AAA), but its invasiveness contributes to substantial perioperative risk. Although Enhanced Recovery After Surgery (ERAS) pathways have improved outcomes across several surgical specialties, evidence supporting their use in open aortic surgery, particularly in Chinese clinical settings, remains limited. We, therefore, evaluated the association between adherence to a locally adapted ERAS pathway and early perioperative outcomes after elective OSR for AAA. **Methods:** This single-center retrospective cohort study included 182 patients who underwent elective OSR for AAA. Patients who received at least 70% of the 30 ERAS elements were assigned to the ERAS group (*n* = 93), whereas those who received less than 70% were assigned to the control group (*n* = 89). A total of 152 patients remained after 1:1 matching of the two groups using propensity score. Quantile regression and logistic regression models were used to evaluate the impact of the ERAS protocol on postoperative length of stay, 30-day mortality, ICU admission rate, hospital cost, major complications, and readmission. **Results:** After matching, baseline and aneurysm characteristics were generally comparable between groups. The ERAS group demonstrated a significantly reduced risk of major complications (OR = 0.33; 95% CI 0.16–0.71; *p* = 0.004) and postoperative nausea and vomiting (OR = 0.10; 95% CI 0.01–0.80; *p* = 0.030). The time to postoperative bowel movement was 1 day earlier in the ERAS group (*p* < 0.001). The incidence of postoperative cardiac complications was significantly lower in the ERAS group (2.6% vs. 11.8%; *p* = 0.028). Pulmonary complications were also markedly reduced in the ERAS group (1.3% vs. 19.7%; *p* < 0.001). The ERAS group was associated with a reduction in postoperative length of hospital stay by 2 days (*p* < 0.001) and a decrease in hospital cost by 8065 RMB (*p* < 0.001). **Conclusions:** Higher adherence to a locally adapted ERAS pathway was associated with fewer major complications, faster bowel recovery, shorter postoperative hospitalization, and lower hospital costs after elective open AAA repair. These findings support prospective multicenter evaluation and further context-specific implementation of ERAS in open aortic surgery.

## 1. Introduction

Abdominal aortic aneurysm (AAA) is a common disease in the field of vascular surgery. Although its prevalence and incidence have declined over the past two decades in many developed countries, rates may be increasing in high-income Asia-Pacific countries and Latin American countries [1]. The two principal treatment strategies are open surgery repair (OSR) and endovascular aneurysm repair (EVAR) [2]. Compared with EVAR, OSR may provide greater long-term durability, with lower rates of all-cause mortality, reintervention, and secondary rupture, making it particularly relevant for patients with complex anatomy, long life expectancy, or failed EVAR. This durability, however, comes at the cost of greater surgical trauma and higher perioperative morbidity and early mortality [3]. Thus, despite rapid advances in endovascular treatment, improving perioperative care for patients who require OSR remains clinically important.

Enhanced Recovery After Surgery (ERAS) is a structured, multimodal approach to perioperative care that aims to attenuate surgical stress and maintain physiologic homeostasis, thereby reducing postoperative complications and length of stay (LOS) [4]. Since Henrik Kehlet first introduced the concept in the late 1990s, ERAS pathways have been adopted widely and have demonstrated both clinical and economic value [5]. ERAS requires the implementation of a patient-centered multidisciplinary team, including surgeons, anesthesiologists, nurses, dietitians and rehabilitation therapists [6]. Although ERAS was initially studied in cardiac and colorectal surgery, its principles have since been extended to many surgical subspecialties [7].

Across surgical specialties, adherence to ERAS pathways has been associated with shorter hospitalization, fewer complications, and lower costs [8]. Adoption in vascular surgery, however, has been comparatively limited. In 2022, the Society for Vascular Surgery and the ERAS Society convened a multidisciplinary expert panel and published evidence-based recommendations for perioperative care in open aortic surgery [9]. Nevertheless, evidence regarding implementation of these recommendations in Chinese clinical settings remains scarce. Accordingly, this study aimed to evaluate whether higher adherence (≥70%) to a locally adapted 30-item ERAS pathway was associated with improved early perioperative outcomes in patients undergoing elective open surgical repair for abdominal aortic aneurysm.

## 2. Materials and Methods

### 2.1. Study Design and Patient Selection

This single-center retrospective cohort study included clinical data from 182 patients with AAA who underwent elective OSR in the Department of Vascular Surgery at Nanjing Drum Tower Hospital between January 2019 and March 2025.

Patients were eligible for inclusion if they met all of the following criteria: (1) AAA confirmed by abdominal aortic computed tomography angiography after admission; (2) fulfillment of the clinical indications for surgical intervention; and (3) elective OSR. Patients were excluded if they met any of the following criteria: (1) infectious AAA; (2) ruptured AAA; (3) emergency surgery; (4) concomitant major aortic disease, including thoracic aortic aneurysm or thoracoabdominal aortic dissection; (5) a cognitive or psychiatric condition that precluded compliance or cooperation with perioperative management; or (6) incomplete clinical data. Based on previous ERAS studies and resources from the ERAS Society, a compliance threshold of ≥70% was set as the criterion for receiving the ERAS pathway [10]. Accordingly, patients who achieved at least 70% of the pathway elements were assigned to the ERAS group, whereas those who achieved less than 70% were assigned to the control group. Propensity score matching was subsequently performed at a 1:1 ratio. This study was approved by the Ethics Committee of Nanjing Drum Tower Hospital, and all patients provided written informed consent before surgery.

### 2.2. Data Collection and Definitions

Basic information of the study subjects was collected through the electronic medical record system, including sex, age, and body mass index (BMI). Recorded comorbidities included hypertension, diabetes, myocardial disease, chronic obstructive pulmonary disease (COPD), hyperlipidemia, chronic kidney disease, cerebral vascular disease, arrhythmia and previous abdominal surgery. All patients underwent abdominal aortic computed tomography angiography after admission. Imaging was used to determine aneurysm diameter and location and to assess renal and iliac artery involvement. AAA was categorized according to its anatomical relationship to the renal arteries as infrarenal or juxtarenal. Juxtarenal AAA was defined as an aneurysm extending to the level of the renal arteries without involvement of the visceral aortic segment. None of the juxtarenal cases included in this study required aortic cross-clamping above the superior mesenteric artery. Operative variables included the methods of arterial anastomosis during OSR (aorto-aortic, aorto-iliac, aortobifemoral, aorto-iliac + aorto-femoral), intraoperative blood loss, intraoperative blood transfusion, and operative time [11].

### 2.3. Adaptation and Implementation of the ERAS Protocol

The pathway used in this study was derived from the 2022 joint consensus statement issued by the Enhanced Recovery After Surgery (ERAS) Society and the Society for Vascular Surgery [9]. The source consensus was developed by an international multidisciplinary panel and included 36 recommendations organized into preadmission, preoperative, intraoperative, and postoperative phases. The original English-language recommendations were reviewed by clinicians experienced in reading English medical literature, and their clinical meaning and local applicability were discussed by a multidisciplinary team comprising vascular surgeons, anesthesiologists, rehabilitation practitioners, nursing staff, and nutritional support staff at Nanjing Drum Tower Hospital.

**Figure 1:**
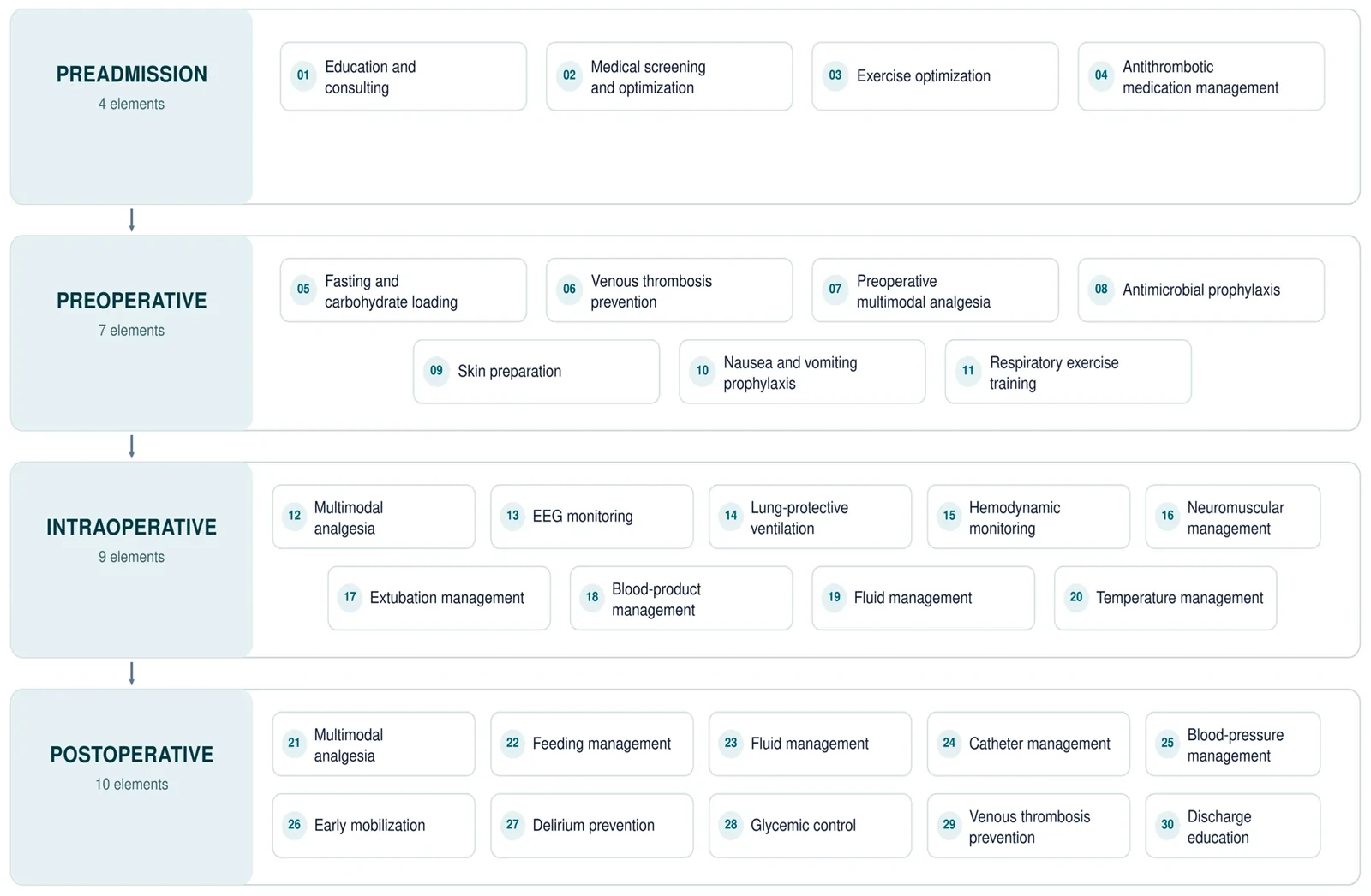
Overview of the locally adapted ERAS pathway for elective open abdominal aortic aneurysm repair. The pathway comprised 30 elements across four perioperative phases: preadmission, preoperative, intraoperative, and postoperative care. Detailed operational definitions of individual elements are provided in Supplementary Table S1.

Each recommendation was evaluated according to its clinical relevance, feasibility, availability of medications and equipment, compatibility with existing hospital workflows, and safety for the local patient population. Recommendations addressing overlapping components of care were combined, and operational targets were adjusted where necessary while preserving the core intent and four-phase structure of the original consensus. This process resulted in the 30-item pathway shown in Figure 1. Because the source document was a clinical consensus rather than a patient-reported instrument or diagnostic scale, no formal psychometric validation was undertaken. The present study represents a preliminary clinical evaluation of the locally adapted pathway rather than formal external validation.

Responsibilities were assigned according to the phase of care. Vascular surgeons and ward nurses were primarily responsible for preadmission and preoperative education, screening, medical optimization, medication management, and preparation. Anesthesiologists, vascular surgeons, and operating-room nurses implemented the intraoperative anesthesia, ventilation, hemodynamic, fluid, temperature, transfusion, neuromuscular blockade, and extubation-related elements. Postoperative management was jointly undertaken by vascular surgeons, ward or intensive-care nurses, rehabilitation practitioners, and nutritional-support staff. No external ERAS certification was required. Protocol adoption was supported by multidisciplinary meetings, departmental teaching, and role-specific briefings covering item-specific targets, staff responsibilities, safety criteria, and clinical documentation.

### 2.4. Assessment of ERAS Compliance

ERAS compliance was assessed retrospectively using a standardized 30-item data-extraction checklist developed from the pathway shown in Figure 1. For details on the 30 ERAS paths, please refer to Supplementary Table S1. The compliance data were not generated automatically by the electronic medical record system. Instead, relevant information was manually extracted from inpatient medical records, medical orders and progress notes, nursing records, anesthesia records, and nutritional screening records.

For each patient, every ERAS element was coded as 1 when its implementation was explicitly documented in at least one relevant source and as 0 when it was not implemented or when no supporting documentation was identified. All elements were assigned equal weight. Patient-level compliance was calculated as follows: ERAS compliance (%) = number of documented elements achieved/30 × 100. Compliance of ≥70%, corresponding to the achievement of at least 21 of the 30 elements, was used to define the ERAS group; patients achieving fewer than 21 elements were assigned to the control group. Element-level adherence was calculated as the number of patients with documented implementation of the corresponding element divided by the total number of patients in each group.

### 2.5. Clinical Outcomes

The primary outcomes were postoperative LOS, early mortality, and readmission. Secondary outcomes included major complications, postoperative ICU admission, hospital cost, time to postoperative bowel movement, postoperative blood transfusion, nausea and vomiting. Early mortality was defined as all-cause death during the index hospitalization or within 30 days and readmission was defined as readmission within 30 days after discharge. Major complications were categorized into nine types, specifically: acute kidney injury requiring hemodialysis, postoperative bleeding requiring reoperation, limb ischemia requiring unplanned intervention, mesenteric ischemia (resulting in death or requiring bowel resection), spinal cord ischemia, major cardiac events (clinically confirmed myocardial infarction or new-onset arrhythmia requiring treatment), major pulmonary complications (ventilation for 48 h or requiring reintubation), chylous fistula and acute stroke. The definitions of the above clinical outcome measures were all based on relevant guidelines from the Society for Vascular Surgery [12].

### 2.6. Statistical Analysis

Normally distributed continuous variables are reported as means with standard deviations, whereas nonnormally distributed variables are reported as medians with interquartile ranges. Categorical variables are reported as frequencies and percentages. Between-group comparisons were performed with the independent-samples *t*-test or Mann–Whitney U test for continuous variables, as appropriate, and Pearson’s chi-square test or Fisher’s exact test for categorical variables. Median quantile regression was used to evaluate associations between ERAS adherence and continuous outcomes, including postoperative LOS, time to postoperative bowel movement, and hospital cost, because these variables were nonnormally distributed. Binary logistic regression was used for early mortality, readmission, major complications, postoperative ICU admission, postoperative blood transfusion, and nausea and vomiting. Regression coefficients with standard errors and odds ratios (ORs) with 95% confidence intervals (CIs) are reported for continuous and binary outcomes, respectively. Propensity scores were estimated from age, sex, BMI, and aneurysm diameter, followed by 1:1 nearest-neighbor matching with a caliper of 0.05. To assess potential temporal confounding, calendar year of surgery was compared between the ERAS and control groups as both a categorical and an ordinal variable. In a sensitivity analysis, propensity-score matching was repeated after adding calendar year to the original model, using 1:1 nearest-neighbor matching. Covariate balance was considered acceptable at an absolute standardized mean difference of <0.10, and the principal outcomes were reanalyzed in the rematched cohort. Two-tailed tests were performed, and the significance level was set at α = 0.05. IBM SPSS Statistics version 27.0 for Windows was used for statistical analysis.

## 3. Results

### 3.1. Patient and Lesion Characteristics

Among the 182 included patients, 93 met the adherence threshold for the ERAS group and 89 were assigned to the control group. After 1:1 propensity-score matching, 76 patients remained in each group. The matched groups were generally comparable with respect to age, sex, BMI, underlying diseases, previous abdominal surgery, AAA size, AAA location, anastomosis, blood loss, and blood transfusion (all *p* > 0.05). Operative time was shorter in the ERAS group than in the control group (213 min vs. 245 min; *p* < 0.001). Baseline and operative characteristics are summarized in Table 1, Table 2 and Table 3.

### 3.2. Primary and Secondary Outcomes

In the matched cohort, the ERAS group had a shorter median postoperative LOS (7 days vs. 9 days; *p* < 0.001), lower median hospital cost (RMB 60,233 vs. 68,294; *p* < 0.001), and earlier postoperative bowel movement (2 days vs. 3 days; *p* < 0.001) than the control group. The incidence of major complications was also lower in the ERAS group (17.1% vs. 38.2%; *p* = 0.004), as was the incidence of postoperative nausea and vomiting (1.3% vs. 11.8%; *p* = 0.009). By contrast, no statistically significant differences were observed in early mortality (1.3% vs. 5.3%; *p* = 0.363), readmission (6.6% vs. 7.9%; *p* = 0.754), postoperative ICU admission (61.8% vs. 73.7%; *p* = 0.118), or postoperative blood transfusion (19.7% vs. 26.3%; *p* = 0.335). These outcomes are summarized in Table 4.

### 3.3. Major Complications

When individual major complications were examined, cardiac complications were less frequent in the ERAS group than in the control group (2.6% vs. 11.8%; *p* = 0.028), as were pulmonary complications (1.3% vs. 19.7%; *p* < 0.001). No statistically significant between-group differences were observed in acute kidney injury requiring hemodialysis (7.9% vs. 15.8%; *p* = 0.132), postoperative bleeding (2.6% vs. 3.9%; *p* > 0.999), limb ischemia (3.9% vs. 2.6%; *p* > 0.999), bowel ischemia (1.3% vs. 5.3%; *p* = 0.363), spinal cord ischemia (1.3% vs. 1.3%; *p* > 0.999), major stroke (1.3% vs. 2.6%; *p* > 0.999), chylous fistula (0% vs. 1.3%; *p* > 0.999). Details are shown in Table 5.

### 3.4. Correlation Analysis of ERAS Protocol and Clinical Outcomes

In logistic regression analyses, adherence to at least 70% of the ERAS pathway was associated with lower odds of major complications (OR, 0.33; 95% CI, 0.16–0.71; *p* = 0.004) and postoperative nausea and vomiting (OR, 0.10; 95% CI, 0.01–0.80; *p* = 0.030). No statistically significant associations were observed for early mortality (OR, 0.24; 95% CI, 0.03–2.20; *p* = 0.207), readmission (OR, 0.82; 95% CI, 0.24–2.82; *p* = 0.755), postoperative ICU admission (OR, 0.58; 95% CI, 0.29–1.15; *p* = 0.120), or postoperative blood transfusion (OR, 0.69; 95% CI, 0.32–1.47; *p* = 0.337).

Median quantile regression showed that ERAS adherence was associated with a 2.0-day reduction in postoperative LOS (*p* < 0.001), a 1.0-day reduction in time to postoperative bowel movement (*p* < 0.001), and an RMB 8065 reduction in hospital cost (*p* < 0.001). The regression results are presented in Table 6.

### 3.5. Compliance

By definition, all patients in the ERAS group achieved at least 70% overall pathway adherence. Element-level adherence nevertheless varied: 17 of the 30 elements (56.7%) were implemented in more than 90% of patients in the ERAS group, whereas several elements remained difficult to implement. In particular, adherence was relatively low for preoperative carbohydrate loading (55.3% in the ERAS group vs. 7.9% in the control group; *p* < 0.001), use of sugammadex for reversal of neuromuscular blockade (26.3% vs. 2.6%; *p* < 0.001), and extubation in the operating room (9.2% vs. 1.3%; *p* = 0.069). Details are shown in Table 7.

### 3.6. Carbohydrate Loading and Perioperative Glucose

Postoperative glucose levels were comparable between the ERAS and control groups on postoperative day (POD) 0 (8.72 mmol/L vs. 9.01 mmol/L; *p* = 0.315), POD 1 (8.09 mmol/L vs. 8.34 mmol/L; *p* = 0.436), and POD 3 (6.90 mmol/L vs. 6.93 mmol/L; *p* = 0.924). A subgroup analysis was then performed within the ERAS group. Patients who received preoperative carbohydrate loading had higher preoperative glucose levels than those who did not (9.50 mmol/L vs. 6.45 mmol/L; *p* < 0.001); however, the two subgroups had similar glucose levels on POD 0 (8.61 mmol/L vs. 8.86 mmol/L; *p* = 0.536), POD 1 (8.02 mmol/L vs. 8.17 mmol/L; *p* = 0.748), and POD 3 (6.87 mmol/L vs. 6.94 mmol/L; *p* = 0.878). Detailed results are shown in Table 8 and Table 9.

### 3.7. Intraoperative Fluid Management

Compared with the control group, the ERAS group received lower median volumes of lactated Ringer’s solution (1123 mL vs. 2328 mL; *p* < 0.001) and hydroxyethyl starch (882 mL vs. 2142 mL; *p* < 0.001). Consequently, the median total intraoperative fluid volume was 40.7% lower in the ERAS group. Fluid-management data are presented in Table 10.

### 3.8. Distribution of Treatment Year and Sensitivity Analysis

Patients in the ERAS group were numerically more likely to have undergone surgery during the later years of the study period; however, the overall calendar-year distribution did not differ significantly between the control and ERAS groups when analyzed as a categorical variable (Pearson chi-square = 3.298; df = 6; *p* = 0.771) or as an ordinal variable (Mann–Whitney U = 4762.5; *p* = 0.076). The mean calendar year was 2021.73 in the control group and 2022.26 in the ERAS group, corresponding to a standardized mean difference of 0.265. After calendar year was added to the propensity-score model, the direction and magnitude of the principal findings remained broadly consistent. Detailed results are provided in Supplementary Table S2.

## 4. Discussion

Open AAA repair remains a high-risk procedure because of the physiologic burden of aortic cross-clamping, the magnitude of the operation, and the comorbidity profile of the patients selected for surgery [13]. The operative complexity and perioperative course may also vary according to aneurysm anatomy, with complex AAA, such as juxtarenal AAA, generally posing greater technical and perioperative challenges than standard infrarenal AAA. Early mortality after open repair of complex AAA has been reported to range from 4.6% to 14.7% [14]. Although endovascular technology has expanded rapidly, OSR remains important for younger patients and for those with complex anatomy, failed EVAR, or a long life expectancy because it may offer greater durability and a lower need for reintervention [15]. As surgical techniques for OSR have reached a bottleneck period with few major breakthroughs [16], perioperative management represents an important opportunity to improve early outcomes. In this context, our study evaluated a locally adapted, multidisciplinary ERAS pathway for elective open AAA repair.

In the propensity-score-matched cohort, adherence to at least 70% of the ERAS pathway was associated with shorter postoperative hospitalization, earlier bowel recovery, lower hospital costs, and fewer major complications, particularly cardiac and pulmonary complications. ERAS adherence was also associated with a lower incidence of postoperative nausea and vomiting, whereas early mortality, readmission, postoperative ICU admission, and blood transfusion did not differ significantly between groups. The direction and magnitude of the principal findings remained broadly consistent after calendar year was incorporated into the propensity-score model. Taken together, these results suggest that a context-specific ERAS pathway may improve early recovery and resource use after open AAA repair. In high-volume Chinese centers, where bed capacity and treatment costs are important practical constraints, the observed 2-day reduction in postoperative LOS and RMB 8065 reduction in median hospital cost may be particularly relevant.

The lower incidence of cardiac and pulmonary complications in the ERAS group may partly reflect differences in perioperative fluid management [17]. Median total intraoperative fluid volume was 40.7% lower in the ERAS group, with reductions in both crystalloid and colloid administration. Excessive perioperative fluid administration can increase cardiac filling pressures, promote ventricular dilation and atrial stretch [18], and contribute to heart failure [19]. Similarly, limiting unnecessary fluid loading may reduce pulmonary vascular hydrostatic pressure and interstitial or alveolar edema [20], thereby improving oxygenation and reducing postoperative pulmonary complications [21].

Earlier bowel recovery and the lower incidence of postoperative nausea and vomiting are also consistent with several linked components of the ERAS pathway [22]. Preoperative carbohydrate loading may attenuate metabolic stress and insulin resistance while helping to preserve intestinal perfusion [23]. In parallel, early postoperative feeding stimulates gastrointestinal motility and digestive secretions, supports the intestinal mucosal barrier, and facilitates nutrient absorption [24]. Rather than acting independently, these elements are likely to contribute collectively to earlier return of gastrointestinal function.

Because concerns about perioperative hyperglycemia can limit implementation of carbohydrate loading, we also examined postoperative glucose levels. Previous studies have generally found carbohydrate loading to be safe and potentially beneficial in selected surgical populations [25,26]. In our cohort, postoperative glucose levels were similar between the ERAS and control groups on POD 0, 1, and 3. Within the ERAS group, patients who received carbohydrate loading had higher preoperative glucose levels than those who did not, but postoperative levels remained comparable. Although our findings suggest that carbohydrate loading does not induce marked hyperglycemia, robust evidence regarding the optimal mode of administration and the appropriate target population for preoperative carbohydrate loading remains lacking. Further studies are needed to evaluate its safety and rationale.

Despite the overall associations with improved recovery, element-level adherence revealed several practical barriers to implementation. Preoperative carbohydrate loading remained underused, partly because of uncertainty regarding patient selection and safety [27]. Use of sugammadex was also limited because anesthesiologists at our center generally used cisatracurium rather than rocuronium or vecuronium for neuromuscular blockade, reducing the applicability of sugammadex-based reversal [28]. In addition, the stringent criteria for extubation after major abdominal surgery meant that relatively few patients were extubated in the operating room [29]. These examples illustrate why implementation success should be assessed at the level of individual pathway components rather than inferred from the availability of a written protocol alone.

Our experience indicates that adapting an ERAS pathway to a non-native English-speaking healthcare environment should be regarded as a structured clinical implementation process rather than as a literal translation of the source recommendations. Such adaptation requires bilingual interpretation of the clinical meaning of each recommendation, multidisciplinary assessment of local feasibility, mapping of recommendations to available medications, equipment, staffing, and documentation systems, explicit assignment of responsibilities across perioperative phases, and continuous audit of element-level adherence and clinical outcomes. Importantly, the core principles of ERAS should be retained, whereas operational details may require modification according to local practice and patient safety. The relatively low adherence to carbohydrate loading, sugammadex use, and extubation in the operating room in our cohort illustrates that recommendations cannot always be transferred unchanged between healthcare systems. Other institutions seeking to implement ERAS in a different linguistic or medical environment should therefore pilot the adapted pathway, provide role-specific staff education, prospectively monitor adherence, and revise locally impractical elements before wider implementation. The present results provide a preliminary clinical evaluation of this adapted pathway; prospective multicenter studies are required for formal external validation.

### Study Limitations

This study has several limitations. First, as a retrospective cohort study, it may be subject to selection bias. Second, the sample size of this study is limited (182 patients). Due to limited statistical power, multivariate adjustment was not performed to avoid model overfitting and resultant instability of the results. For low-incidence outcomes such as early mortality, the statistical power is even more insufficient, and the relevant results should be interpreted with caution. Furthermore, although patient grouping in this study was based on actual adherence to the ERAS protocol rather than simply on chronological order, time-related confounding factors, such as improvements in surgical proficiency and cumulative experience in ERAS management, may still exist. Although the calendar-year distribution did not differ significantly between the two groups, patients in the ERAS group were numerically more likely to have been treated during the later years. Consequently, potential temporal confounding cannot be completely excluded. Improvements in refinement of anesthetic and perioperative management, and the accumulation of institutional experience in implementing the ERAS pathway may have contributed to improved outcomes over time. Although the principal findings remained broadly consistent after adjustment for calendar year, residual confounding arising from unmeasured secular changes may persist. Future prospective, multicenter studies with contemporaneous comparison groups and standardized ERAS implementation are warranted.

## 5. Conclusions

Among patients undergoing elective OSR for AAA, adherence to at least 70% of a locally adapted ERAS pathway was associated with fewer major complications, faster gastrointestinal recovery, shorter postoperative hospitalization, and lower hospital costs. Because the retrospective design does not establish causality, these findings should be considered hypothesis-generating and require prospective multicenter validation. Nevertheless, they support the feasibility and potential value of context-specific ERAS implementation in Chinese vascular surgery centers.

## Figures and Tables

**Table 1:** Baseline characteristics of patients before propensity score matching.

Patient Characteristics	Control Group (*n* = 89)	ERAS Group (*n* = 93)	*p*-Value
Age (years)	63 (60–68)	66 (61–69)	0.057
Male gender	77 (86.5)	81 (87.1)	0.908
BMI (kg/m^2^)	23 (21–24)	23 (21–25)	0.159
Hypertension	50 (56.2)	61 (65.6)	0.193
Diabetes	14 (15.7)	17 (18.3)	0.647
Myocardial disease	16 (18)	19 (20.4)	0.675
COPD	6 (6.7)	10 (10.8)	0.339
Hyperlipidemia	9 (10.1)	9 (9.7)	0.922
Chronic kidney disease	2 (2.2)	3 (3.2)	0.686
Cerebral vascular disease	10 (11.2)	10 (10.8)	0.917
Arrhythmia	8 (9)	9 (9.7)	0.873
Prior abdominal surgery	3 (3.4)	10 (10.8)	0.053
AAA size (mm)	59 (52–75)	61 (50–70)	0.944
AAA location			0.198
Infrarenal involved iliac artery	57 (64)	50 (53.8)	
Infrarenal not involved iliac artery	32 (36)	40 (43)	
Juxtarenal involved iliac artery	0 (0)	2 (2.2)	
Juxtarenal not involved iliac artery	0 (0)	1 (1.1)	

AAA, abdominal aortic aneurysm; BMI, body mass index; COPD, chronic obstructive pulmonary disease. Data are presented as number (%) or median (interquartile range).

**Table 2:** Baseline characteristics of patients after propensity score matching.

Patient Characteristics	Control Group (*n* = 76)	ERAS Group (*n* = 76)	*p*-Value
Age (years)	64 (60–69)	65 (61–71)	0.384
Male gender	67 (88.2)	64 (84.2)	0.481
BMI (kg/m^2^)	23 (22–24)	23 (21–24)	0.761
Hypertension	47 (61.8)	49 (64.5)	0.737
Diabetes	13 (17.1)	14 (18.4)	0.832
Myocardial disease	14 (18.4)	15 (19.7)	0.836
COPD	6 (7.9)	8 (10.5)	0.575
Hyperlipidemia	7 (9.2)	8 (10.5)	0.786
Chronic kidney disease	2 (2.6)	3 (3.9)	>0.999
Cerebral vascular disease	9 (11.8)	7 (9.2)	0.597
Arrhythmia	7 (9.2)	8 (10.5)	0.786
Prior abdominal surgery	3 (3.9)	7 (9.2)	0.191
AAA size (mm)	59 (50–71)	61 (50–70)	0.419
AAA location			0.242
Infrarenal involved iliac artery	51 (67.1)	43 (56.6)	
Infrarenal not involved iliac artery	25 (32.9)	32 (42.1)	
Juxtarenal involved iliac artery	0 (0)	1 (1.3)	
Juxtarenal not involved iliac artery	0 (0)	0 (0)	

AAA, abdominal aortic aneurysm; BMI, body mass index; COPD, chronic obstructive pulmonary disease. Data are presented as number (%) or median (interquartile range).

**Table 3:** Comparison of surgical details.

Details	Control Group (*n* = 76)	ERAS Group (*n* = 76)	*p*-Value
Anastomosis			0.289
Aorto-aortic	16 (21.1)	24 (31.6)	
Aorto-iliac	53 (69.7)	49 (64.5)	
Aortobifemoral	4 (5.3)	1 (1.3)	
Aorto-iliac and aorto-femoral	3 (3.9)	2 (2.6)	
Blood loss (mL)	1250 (990–2000)	1200 (800–1500)	0.059
Blood transfusion (mL)	1050 (675–1510)	1050 (775–1300)	0.730
Operative time (minutes)	245 (230–278)	213 (200–240)	<0.001

Data are presented as number (%) or median (interquartile range).

**Table 4:** Comparison of primary and secondary outcomes.

Primary and Secondary Outcomes	Control Group (*n* = 76)	ERAS Group (*n* = 76)	*p*-Value
LOS (days)	9 (7–12)	7 (6–9)	<0.001
Hospital cost (RMB)	68,294 (63,198–76,161)	60,233 (56,706–68,199)	<0.001
Postoperative bowel movement (days)	3 (3–3)	2 (2–3)	<0.001
Mortality	4 (5.3)	1 (1.3)	0.363
Readmissions	6 (7.9)	5 (6.6)	0.754
Major complications	29 (38.2)	13 (17.1)	0.004
ICU	56 (73.7)	47 (61.8)	0.118
Postoperative blood transfusion	20 (26.3)	15 (19.7)	0.335
Nausea and vomiting	9 (11.8)	1 (1.3)	0.009

LOS, length of stay; ICU, Intensive Care Unit. Data are presented as number (%) or median (interquartile range).

**Table 5:** Comparison of major complications.

Major Complications	Control Group (*n* = 76)	ERAS Group (*n* = 76)	*p*-Value
AKI-HD	12 (15.8)	6 (7.9)	0.132
Postoperative bleeding	3 (3.9)	2 (2.6)	>0.999
Limb ischemia	2 (2.6)	3 (3.9)	>0.999
Bowel ischemia	4 (5.3)	1 (1.3)	0.363
Spinal cord ischemia	1 (1.3)	1 (1.3)	>0.999
Cardiac complication	9 (11.8)	2 (2.6)	0.028
Pulmonary complication	15 (19.7)	1 (1.3)	<0.001
Major stroke	2 (2.6)	1 (1.3)	>0.999
Chylous fistula	1 (1.3)	0 (0)	>0.999

AKI-HD, acute kidney injury requiring hemodialysis. Data are presented as number (%).

**Table 6:** Effect of ERAS protocol on primary and secondary outcomes.

Outcomes	OR (95% CI)	*p*-Value
Mortality	0.24 (0.03–2.20)	0.207
Readmissions	0.82 (0.24–2.82)	0.755
Major complications	0.33 (0.16–0.71)	0.004
ICU	0.58 (0.29–1.15)	0.120
Postoperative blood transfusion	0.69 (0.32–1.47)	0.337
Nausea and vomiting	0.10 (0.01–0.80)	0.030
**Outcomes**	**Effect (SE)**	***p*** **-Value**
LOS (days)	−2.0 (0.51)	<0.001
Hospital cost (RMB)	−8065 (1960)	<0.001
Postoperative bowel movement (days)	−1.0 (0.17)	<0.001

CI, confidence interval; OR, odds ratio; SE, standard error.

**Table 7:** Compliance with ERAS pathways.

Details	Control Group (*n* = 76)	ERAS Group (*n* = 76)	*p*-Value
Preadmission			
Educationandconsulting	31 (40.8)	74 (97.4)	<0.001
Medical screening and optimization	35 (46.1)	73 (96.1)	<0.001
Exercise optimization	36 (47.4)	73 (96.1)	<0.001
Anticoagulation	33 (43.4)	68 (89.5)	<0.001
Preoperative			
Fasting management	6 (7.9)	42 (55.3)	<0.001
Venous thrombosis prevention	29 (38.2)	71 (93.4)	<0.001
Preoperative multimodal analgesia	35 (46.1)	70 (92.1)	<0.001
Anti-infection	51 (67.1)	71 (93.4)	<0.001
Skin preparation	73 (96.1)	75 (98.7)	0.612
Prevention of nausea and vomiting	31 (40.8)	72 (94.7)	<0.001
Respiratory exercise training	33 (43.4)	73 (96.1)	<0.001
Intraoperative			
Intraoperative multimodal analgesia	24 (31.6)	60 (78.9)	<0.001
EEG monitoring	25 (32.9)	68 (89.5)	<0.001
Lung-protective ventilation	32 (42.1)	73 (96.1)	<0.001
Hemodynamic monitoring	30 (39.5)	71 (93.4)	<0.001
Neuromuscular management	2 (2.6)	20 (26.3)	<0.001
Tracheal intubation management	1 (1.3)	7 (9.2)	0.069
Blood products	19 (25)	60 (78.9)	<0.001
Fluid management	23 (30.3)	67 (88.2)	<0.001
Temperature management	73 (96.1)	75 (98.7)	0.612
Postoperative			
Postoperative multimodal analgesia	19 (25)	63 (82.9)	<0.001
Feeding management	18 (23.7)	46 (60.5)	<0.001
Fluid management	23 (30.3)	67 (88.2)	<0.001
Catheter management	32 (42.1)	73 (96.1)	<0.001
Blood pressure management	24 (31.6)	57 (75)	<0.001
Mobilization	31 (40.8)	73 (96.1)	<0.001
Delirium prevention	34 (44.7)	75 (98.7)	<0.001
Glycemic control	35 (46.1)	63 (82.9)	<0.001
Prevention of deep vein thrombosis	71 (93.4)	75 (98.7)	0.211
Discharge education	32 (42.1)	75 (98.7)	<0.001

EEG, electroencephalogram. Data are presented as number (%).

**Table 8:** Comparison of perioperative glucose.

Perioperative Glucose	Control Group (*n* = 76)	ERAS Group (*n* = 76)	*p*-Value
POD0 glucose (mmol/L)	9.01 ± 1.77	8.72 ± 1.70	0.315
POD1 glucose (mmol/L)	8.34 ± 1.97	8.09 ± 2.00	0.436
POD3 glucose (mmol/L)	6.93 ± 1.98	6.90 ± 2.05	0.924

POD, postoperative day. Data are presented as mean ± standard deviation.

**Table 9:** Subgroup analysis of perioperative glucose in ERAS group.

Perioperative Glucose	No Carbohydrate Loading (*n* = 34)	Carbohydrate Loading (*n* = 42)	*p*-Value
Pre-Op glucose (mmol/L)	6.45 ± 1.85	9.50 ± 2.69	<0.001
POD0 glucose (mmol/L)	8.86 ± 1.70	8.61 ± 1.71	0.536
POD1 glucose (mmol/L)	8.17 ± 2.03	8.02 ± 2.00	0.748
POD3 glucose (mmol/L)	6.94 ± 2.01	6.87 ± 2.10	0.878

POD, postoperative day. Data are presented as mean ± standard deviation.

**Table 10:** Comparison of intraoperative fluid management.

Project	Control Group (*n* = 76)	ERAS Group (*n* = 76)	*p*-Value
LR (mL)	2328 (2024–3657)	1123 (712–1424)	<0.001
HES (mL)	2142 (1475–2573)	882 (502–1324)	<0.001
Total volume infused (mL)	5546 (4178–7389)	3289 (2178–4389)	<0.001

HES, hydroxyethyl starch; LR, lactated Ringer’s solution. Data are presented as median (interquartile range).

## Data Availability

The data and materials can be obtained by contacting the corresponding author.

## Supplementary Materials

The following supporting information can be downloaded at: https://www.mdpi.com/article/10.3390/jcm15166486/s1, Table S1: ERAS clinical pathway protocol; Table S2: Distribution of treatment year and sensitivity analysis.
